# Exercise-Mediated Browning of White Adipose Tissue: Its Significance, Mechanism and Effectiveness

**DOI:** 10.3390/ijms222111512

**Published:** 2021-10-26

**Authors:** Wang-Jing Mu, Jie-Ying Zhu, Min Chen, Liang Guo

**Affiliations:** 1 School of Kinesiology, Shanghai University of Sport, Shanghai 200438, China; muwangjing0502@163.com (W.-J.M.); zhujieying1127@163.com (J.-Y.Z.); cminfinite923@163.com (M.C.); 2 Shanghai Frontiers Science Research Base of Exercise and Metabolic Health, Shanghai University of Sport, Shanghai 200438, China

**Keywords:** browning of WAT, exercise, Ucp1, ROS, metabolites, exerkines, SNS, lipolysis

## Abstract

As a metabolic organ, adipose tissue plays an important role in regulating metabolism. In adults, most adipose tissue is white adipose tissue (WAT), and excessive expansion of WAT will lead to obesity. It is worth noting that exercise can reduce the fat mass. There is also a lot of evidence that exercise can promote the browning of WAT, which is beneficial for metabolic homeostasis. Multiple factors, including reactive oxygen species (ROS), metabolites, nervous system, exerkines and lipolysis can facilitate exercise-mediated browning of WAT. In this review, the roles and the underlying mechanisms of exercise-mediated browning of WAT are summarized. The effects of different styles of exercise on the browning of WAT are also discussed, with the aim to propose better exercise strategies to enhance exercise-mediated browning of WAT, so as to promote metabolic health. Finally, the different reactivity of WAT at different anatomical sites to exercise-mediated browning is reviewed, which may provide potential suggestion for people with different fat loss needs.

## 1. Introduction

In general, mammals have three types of adipose tissue, white adipose tissue (WAT), brown adipose tissue (BAT) and beige adipose tissue. The main function of WAT is to store and mobilize energy, and it is categorized into two types of WAT, the visceral white adipose tissue (vWAT) and the subcutaneous white adipose tissue (scWAT). WAT stores energy in the form of triglycerides and mobilizes energy in the form of fatty acids (FAs) [1]. BAT expends energy for thermogenesis when it is activated, a process known as non-shivering thermogenesis. The energy consumption and thermogenic capacity of BAT are largely related to the expression of uncoupling protein 1(Ucp1), a protein located in the inner membrane of mitochondria. After BAT is activated, Ucp1 mediates the respiratory uncoupling, leading to the generation of heat [2]. Beige adipose tissue, located in WAT, is similar to BAT and can produce heat. The biogenesis of beige adipose tissue can also regulate metabolism. It was shown in [3] that adult WAT can undergo beige adipogenesis, including de novo beige adipocyte differentiation and trans-differentiation from white to beige adipocytes, after some stimulation (such as cold and exercise). The process above is called the browning of WAT. Because WAT accounts for a major part of adipose tissue in the humans after infancy, strategies to promote the browning of WAT could provide a huge potential for improving metabolism and combating obesity. The concept of “exercise is medicine” has been well recognized. Exercise can reduce the fat mass in adipose tissue and improve the whole-body metabolism [4,5,6], in which the browning of WAT is involved. Therefore, exercise-mediated browning of WAT serves as a promising strategy in the fight against obesity and related metabolic disorders.

## 2. The Significance of Exercise-mediated Browning of WAT

Exercise improves metabolic health, with multiple factors involved in this process. Irisin is an important myokine induced by exercise. Exercise promotes the expression of the peroxisome proliferator-activated receptor γ (PPARγ) coactivator-1 α (PGC-1α) in skeletal muscle, which subsequently stimulates the expression of the fibronectin type III domain containing 5 (Fndc5) in skeletal muscle. The cleavage of Fndc5 produces irisin, which is secreted into circulation and reaches adipose tissue. Therefore, a PGC-1α-mediated increase of Fndc5 expression will lead to more irisin production. In the way discussed above, exercise triggers the increase of irisin. Pontus Bostrom et al. [7] conducted exercise programs of three weeks’ free-wheeling exercise in mice and 10 weeks’ endurance exercise in healthy people. Irisin levels in the blood of mice and humans increased after exercise. Pontus Bostrom et al. used adenoviral vectors to over-express full-length Fndc5, which led to elevated plasma levels of irisin in mice. After that, an increase in Ucp1 mRNA and the number of Ucp1 positive cells were observed in the scWAT of mice. In addition, improvement in glucose metabolism and insulin sensitivity were observed after elevating the irisin level in mice. Therefore, irisin is secreted from the skeletal muscle into the circulation and reaches adipose tissue to promote the browning of WAT. How long it takes until the irisin level is increased by exercise and the duration of the up-regulation of irisin by exercise is worthy of further research.

In another study, mice were intraperitoneally injected with an anti-Fncd5 antibody to impair irisin production and secretion. After 10 days of swimming training [8], the sudden decrease of Ucp1 and Cidea gene expression in WAT was observed in mice treated with the anti-Fncd5 antibody. This is due to the impaired production of irisin after the injection of the Fncd5 antibody, resulting in the decreased expression of irisin. Thus, when the expression of irisin decreased, the exercise-mediated browning of WAT was also attenuated. Irisin has been reported to mediate the browning of WAT through Mitogen-Activated protein kinase (P38) and extracellular signal-associated kinase (ERK) signaling [9]. The increase of Ucp1 mediated by irisin was prevented by the inhibition of P38 or ERK. Therefore, exercise leads to the up-regulation of irisin, which is an important hormone for the browning of WAT and the improvement of metabolism.

In addition, exercise increases the plasma level of fibroblast growth factor 21(FGF21). In one study [10], seven healthy men were given an exercise intervention at 60% of their Vo_2max_ for 60 min. After the exercise, the subjects’ venous blood was collected. The study demonstrated that exercise induced an increase in plasma FGF21. In another study, the increase of FGF21 can mediate the browning of WAT to increase energy consumption [11]. FGF21 is secreted mainly by the liver, which regulates the circulating levels of FGF21. In addition, FGF21 can be produced and secreted by adipose tissue. As an autocrine factor, adipose tissue-derived FGF21 up-regulates the expression of Ucp1 in WAT. Thus, exercise can induce FGF21, a well-known browning hormone with beneficial effects on metabolism. Collectively, the examples of FGF21 and irisin confirm the significance of exercise-mediated browning in promoting metabolic health.

Studies have shown that the effect of exercise on improving metabolism was weakened when the effect of WAT browning was weakened. Yan Xiong et al. [8] used transcription activator-like effector nucleasen (TALEN)-mediated DNA-targeting technology to produce Fndc5 mutant mice. The mutant and control mice were then put on a treadmill for two months, trained for one hour a day. The mutant and control mice had the same amount of exercise. They ran for 60 min a day, 5 days a week, for 8 weeks. The running speed was 18 m per minute and the slope was fixed at 10%. The results showed that exercise increased glucose tolerance, insulin sensitivity, maximal oxygen uptake, and Ucp1 mRNA expression in control mice. It suggests that exercise can mediate the browning of WAT and improve metabolism. However, in mice with Fndc5 mutation, exercise-mediated UCP1 mRNA up-regulation was impaired. Meanwhile, the improvement of glucose tolerance, insulin sensitivity and Vo_2max_ was attenuated. Therefore, exercise-mediated metabolic improvement was attenuated when exercise-induced browning of WAT was blocked upon the inactivation of irisin. Furthermore, low temperature is important for the browning of WAT. An experiment [12] was conducted to study the inguinal white adipose tissue (iWAT) browning and insulin tolerance of mice in the sedentary group (SED) and voluntary wheel running group (VWR) at room temperature (RT = 22 °C) and thermoneutrality (TN = 29 °C). Firstly, exercise reduced the lipid drop size of iWAT, and the effect of exercise on reducing lipid droplets was weakened after temperature raised. Secondly, exercise can also promote the increase of Ucp1 expression in iWAT, and the exercise-mediated increase of Ucp1 was also weakened when the temperature increased. Thirdly, exercise improves insulin sensitivity, which is weakened with the rise of temperature. Mechanistically, the temperature-sensitive sympathetic nerve system (SNS) is critical for the browning of WAT. When the temperature is low, the SNS is activated and the browning of WAT is enhanced. At thermoneutrality, however, the browning of WAT is weakened owing to inactivated SNS. Therefore, at temperatures near thermoneutrality, the effect of exercise-mediated browning of WAT is weakened, in the meantime, the effect of exercise on improving metabolism is also attenuated.

Taken together, these studies highlight an important role of exercise-induced browning of WAT in the improvement of metabolism mediated by exercise. As far as we know, there is no research work investigating the difference between obese individuals and non-obese individuals in the responsiveness to exercise-mediated browning of WAT. It is also unclear whether the effect of exercise-mediated browning of WAT depends on the individual’s physiological conditions such as health and disease. However, the points above merits further investigation in the future studies. In addition, to study and compare the effect of exercise on individuals with and without WAT browning, after performing exercise under room temperature, may help to better understand the significance of exercise-mediated WAT browning in the improvement of health conditions.

## 3. Mechanism of Exercise-Mediated Browning of WAT

**Figure 1:**
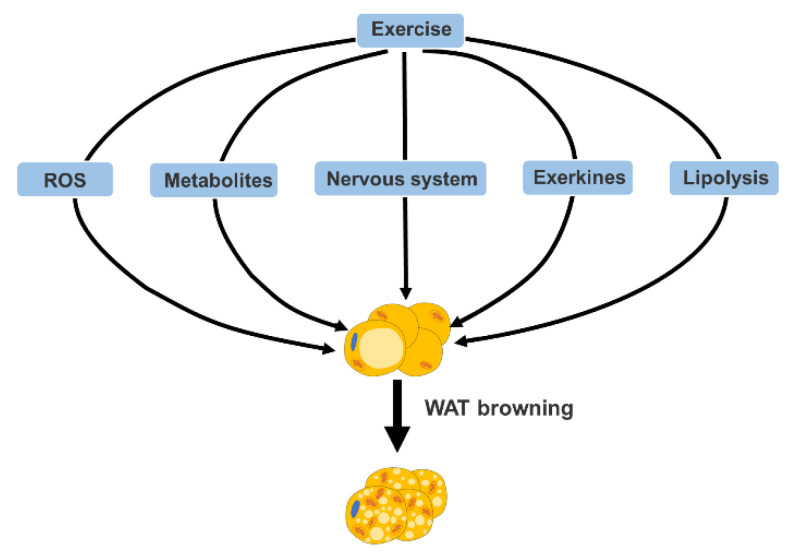
Mechanism of exercise-mediated WAT browning. Multiple factors, including reactive oxygen species (ROS), metabolites, nervous system, exerkines and lipolysis can facilitate exercise-mediated browning of WAT.

Exercise-mediated browning of WAT involves various mechanisms, which will be discussed in this section from the following five aspects: reactive oxygen species (ROS), metabolites, nervous system, exerkines and lipolysis, which are summarized in Figure 1.

### 3.1. Reactive Oxygen Species (ROS)

ROS is involved in various physiological and pathophysiological processes and is well recognized as a double-edged sword. ROS also plays an important role in mediating the browning of WAT. Nuclear factor-erythroid-2-related Transcription factor-2 (Nrf2) is a key regulator of genes involved in the defense against oxidative stress. It was found that [13] Nrf2^-/-^ mice have decreased levels of glutathione (GSH) in WAT. The results showed that Nrf2^-/-^ mice consumed two times more oxygen and had a significantly higher level of ROS compared to wild-type mice. It is worth noting that the mRNA and protein expression of Ucp1 in the WAT of Nrf2^-/-^ mice increased. Another paper also suggested that down-regulation of GSH in WAT drives the browning of WAT [14]. They used cold exposure to induce the browning of WAT and found a decrease in GSH in epididymal white adipose tissue (eWAT), which was accompanied by a decrease in the fat droplets of eWAT and the number of fat cells. In addition, treatment with buthionine sulfoximine (BSO), a GSH inhibitor, resulted in increased expression of Ucp1 in eWAT. GSH provides electrons for glutathione peroxidase (GPXs), which eliminates H_2_O_2_. So, reducing GSH can increase H_2_O_2_ in WAT. It was found [15] that activation of spermidine/spermine N1-acetyltransferase (SAT1) can induce ROS to promote browning of white adipocytes and heat production. Primary precursor adipocytes were isolated from iWAT and differentiated into adipocytes. After the adipocytes were treated with N1, N11-Diethylnorspermin (DENSPM), SAT1 was activated, and the levels of H_2_O_2_ increased, leading to an increase of Ucp1 mRNA expression. Elevated ROS levels induce increased expression of Ucp1, which promotes the browning of white adipocytes. Edward T et al. [16] demonstrated the increased mitochondrial ROS (mtROS) levels and sulfenylation of Ucp1, following acute cold exposure. They also found that changes in mtROS did not affect the expression of heat-producing genes in WAT, but mitochondrial respiration, which was attributable to mtROS-mediated Ucp1 sulfenylation that can enhance Ucp1 activity. Collectively, preventing H_2_O_2_ consumption or increasing H_2_O_2_ levels can increase thermogenic genes expression and facilitate the browning of WAT. Besides, mtROS could enhanced Ucp1 activity by inducing its sulfenylation.

Exercise is known to cause oxidative stress. In recent years, it has been found that exercise-mediated oxidative stress plays a beneficial role in the body. Exercise induces oxidative stress in skeletal muscles. Hypertrophy training is a resistance training. The effects of different resistance exercises on oxidative stress in Wistar rats were examined [17]. The results showed that superoxide dismutase 2 (SOD_2_) activity in the muscles of both the hypertrophy training group and strength training group was significantly higher than that of the non-training group. Therefore, resistance training can increase SOD_2_ activity. Because SOD_2_ can convert O_2_^−^ to H_2_O_2_, the increase of SOD_2_ activity can promote the level of H_2_O_2_. Several reviews [18,19,20] also made a comprehensive summary of exercise-induced oxidative stress, indicating that exercise can cause skeletal muscle oxidative stress and increase ROS, including H_2_O_2_. Since H_2_O_2_ can enter WAT through blood circulation, we propose that exercise promotes the production and release of H_2_O_2_, which may enter WAT through blood circulation and promote the browning of WAT by inducing thermogenic genes expression. Further studies are needed to verify the above hypothesis.

Evanna L. Mills et al. [21] found that treating mice with succinate led to increased ROS levels in adipose tissue. They found that succinate oxidation, via the succinate dehydrogenase (SDH), increases ROS production in adipose tissue. After SDH inhibition, succinate-dependent ROS increase was eliminated. Interestingly, it was found that acute exercise increases succinate levels in skeletal muscles. Anita Reddy et al. [22] examined the tibialis anterior muscle of mice following an acute exercise intervention and found increased succinate levels. Moreover, after 60 min of cycling training, healthy men had increased levels of succinate in their blood. Thus, it is hypothesized that exercise-mediated up-regulation of succinate could stimulate the increase of mtROS in adipose tissue and promote Ucp1-activity by the sulfenylation of Ucp1 (also see the discussion in the next section).

**Figure 2:**
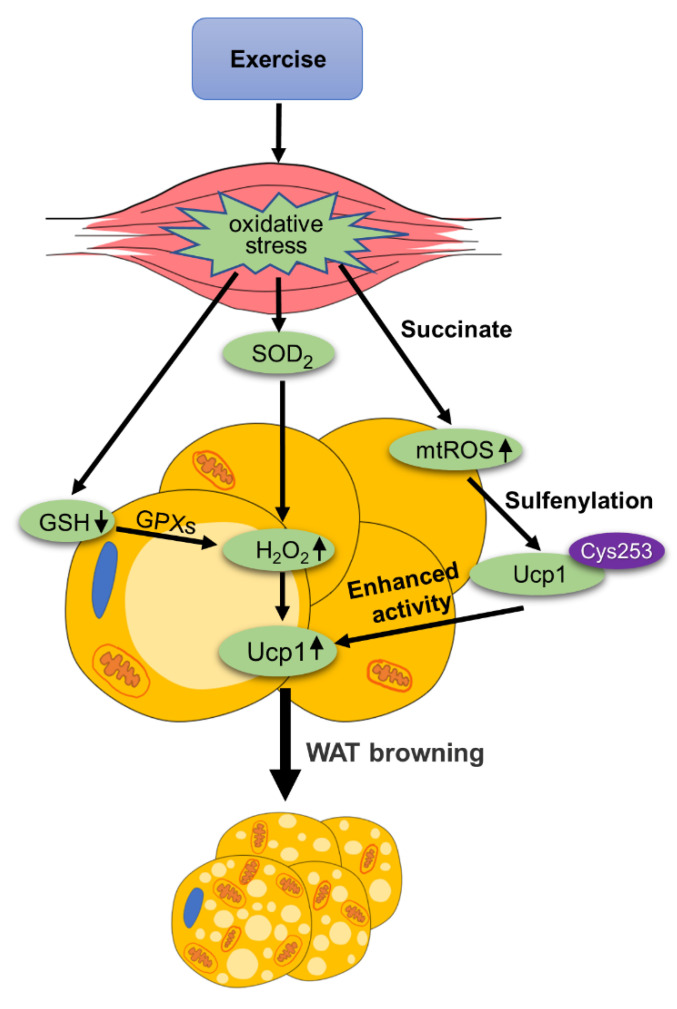
The role of ROS in exercise-mediated WAT browning. Exercise causes oxidative stress in skeletal muscle and reduces the level of GSH. GSH provides electrons for glutathione peroxidase (GPXs), which eliminates H_2_O_2_. Reducing GSH in WAT, can increase H_2_O_2_. Oxidative stress can also enhance superoxide dismutase 2 (SOD_2_) activity, which reduces ROS to H_2_O_2_. H_2_O_2_ enters WAT through blood circulation and mediates the browning of WAT by enhancing the expression of Ucp1. Exercise increases the level of succinate, causing an increase in mitochondrial reactive oxygen species (mtROS) levels. The increase in mtROS promotes Cys253 sulfenylation to enhance the activity of Ucp1.

Therefore, exercise-mediated oxidative stress can produce ROS, which plays an important role in the browning of WAT, as is summarized in Figure 2.

### 3.2. Metabolites

**Figure 3:**
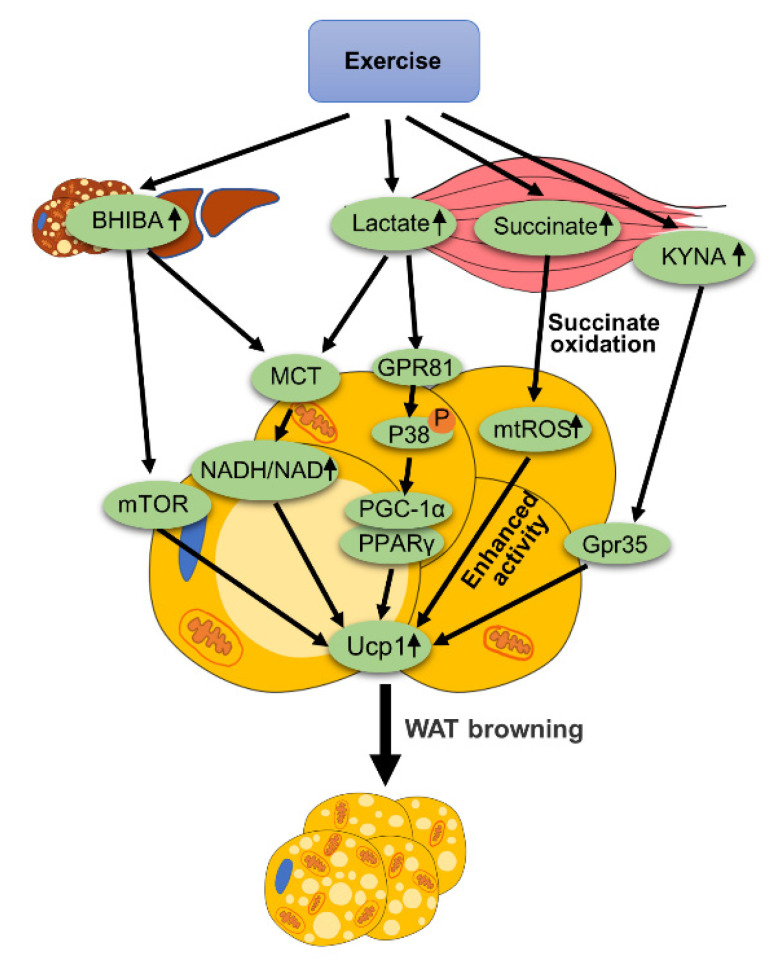
The role of metabolites in exercise-mediated WAT browning. Exercise increases BHIBA levels in the liver and skeletal muscle. BHIBA mediates the browning of WAT through the mTOR signaling pathway or elevated NADH/NAD^+^ ratio. Exercise induces skeletal muscle to produce lactate. Lactate binds to receptor GRP81 on adipocytes, which promotes the phosphorylation of P38, leading to the increased expression of PGC-1α and PPARγ. This process ultimately increases Ucp1 expression. Exercise increases the level of succinate. Oxidation of succinate can increase the mtROS level and enhance the activity of Ucp1. Exercise promotes KYNA release in skeletal muscle, which binds to and activates Gpr35 on adipocytes. This process also leads to WAT browning.

Exercise can mediate the production of many metabolites, such as lactate, ketone bodies, succinate and kynurenic acid (KYNA). These metabolites produced by exercise can lead to the browning of WAT, as is summarized in Figure 3.

#### 3.2.1. Lactate

Zhijie Yao et al. [23] showed that lactate and its receptor GPR81 play an important role in the browning of WAT. β3-adrenergic stimulation up-regulates the expression of GPR81 in adipose tissue and is accompanied by an increase in Ucp1 expression. After an adipose tissue-specific knockout of GPR81, β3-adrenergic agonist-mediated browning of WAT was impaired. Zhijie Yao et al. found that dietary supplementation of lactate can enhance the browning of WAT and reduce obesity. The mechanism of lactate promoting the browning of WAT may be related to P38 MAPK. Previous studies have shown that P38 MAPK mediates browning of WAT by activating PGC-1a, PPARγ and Ucp1 gene expression [24]. Zhijie Yao et al. also found that the mRNA expression of PGC-1α, PPARγ and Ucp1 in mouse eWAT and iWAT increased by using a supplement of lactate, which showed a dose-dependent-manner. The phosphorylation of P38 and the expression of Ucp1 in the adipose tissue of mice were increased when lactate was added into the diet. However, after the pharmacological inhibition of P38 MAPK, the effect of lactate on inducing Ucp1 expression was eliminated. Therefore, we can make a hypothesis that exercise promotes the secretion of lactate from skeletal muscle, and then lactate binds to GPR81 on adipocytes to trigger P38 signaling. Lactate activates the expression of PGC-1α and PPARγ through P38 to mediate WAT browning. It is worth noting that in this experiment, lactate was added to the diet, and its lactate level in WAT was about 7 mmol/L, which was higher than that in a physiological state. We all know that after strenuous exercise, or exercise with insufficient oxygen supply, the skeletal muscle will release a lot of lactates. What intensity of exercise can make lactate in WAT reach a level similar to that in the above experiment? This may be an effective way to realize the browning of WAT via exercise. In addition, the appropriate dose range of lactate, in the aspects of both effectiveness and safety for enhancement of WAT browning, needs further investigation. Another review [25] also reported that adipose tissue absorbs lactate through monocarboxylate transporter (MCT), increasing the NADH/NAD^+^ ratio. Up-regulation of NADH/NAD results in increased redox pressure. This leads to increased Ucp1 expression that can help deplete NADH and release redox pressure, a process that ultimately promotes WAT browning.

Thus, lactate can promote the browning of WAT through the GPR81/P38 MAPK axis or elevating NADH/NAD^+^ ratio, which could be involved in exercise-mediated browning of WAT.

#### 3.2.2. Succinate

It is well known that exercise can induce an increase in the release of succinate [22]. Anita Reddy et al. performed an acute exercise intervention on mice to detect the release level of succinate in mice. The experiment used treadmill exercise until the mice were exhausted to induce muscle remodeling. After separating and examining the tibialis anterior muscle of the mouse, it was found that the level of succinate in the muscle was increased. This has also been confirmed in humans. Experimenters inserted catheters into individuals’ femoral arteries and veins to sample local blood from exercised muscles. The individuals exercised on a bicycle ergometer with 67% of the maximum oxygen uptake for 60 min, and their local blood during exercise and recovery was collected and analyzed. It was found that the level of succinate in the arteries and veins increased. In short, acute exercise can increase succinate levels in humans and mice. Moreover, the author further confirmed that the selective release of succinate in muscle cells is achieved by monocarboxylate transporter 1 (MCT1) through a pH-gated transport. It was found in [21] that compared with 29 ℃ environments, the level of succinate in the scWAT of mice was greatly increased when they were exposed to cold temperatures for 2 weeks. The increase of systemic succinate stimulates Ucp1-dependent heat production in the body and prevents obesity. Another article also confirmed [26] that increasing the quality of beige adipose tissue can promote the absorption of succinate by beige fat cells from body fluids, thereby reducing the concentration of extracellular succinate in the liver to inhibit the progression of liver inflammation and non-alcoholic fatty liver disease (NAFLD). Moreover, without Ucp1, the ability of beige fat to remove succinate from circulation will be reduced. Ucp1-KO mice have elevated levels of succinate in the circulation, which promotes liver inflammation by binding to its receptor succinate receptor 1 (SUCNR1) on hepatocytes. Succinate dehydrogenase mediated-succinate oxidation in adipocytes initiates the production of mtROS and drives thermogenic respiration by enhancing Ucp1 activity in the Ucp1 Cys253 sulfenylation-dependent manner, while succinate dehydrogenase inhibition blunts thermogenic respiration. Thus, the mtROS led to sulfenylation of Ucp1 in Cys253, which enhanced the activity of Ucp1 and promoted heat production and energy consumption.

In conclusion, succinate promotes WAT browning by enhancing Ucp1-dependent thermogenesis and indirectly ameliorates liver inflammation and non-alcoholic steatohepatitis (NASH). Exercise-mediated up-regulation of succinate could facilitate exercise-induced browning of WAT and inhibit the progression of NAFLD.

#### 3.2.3. Ketone Bodies

Ketone bodies are small lipid-derived molecules that provide energy to tissues during glucose deficiency, such as prolonged exercise. The ketone body usually consists of three molecules: beta-hydroxybutyrate (BHIBA), acetoacetic acid and acetone. When the production of ketones is activated, blood levels of BHIBA rise much faster than acetoacetic acid and acetone, and most of the ketones are in the form of BHIBA. BHIBA is mainly produced by the liver. It was found that BHIBA can also be produced and exported from brown fat cells and induces the expression of brown adipocytes-related genes in primary adipocytes [27]. Anna Whitehead et al. induced the browning of primary adipocytes partially differentiated from the stromal vascular fraction (SVF) of scWAT. It was found that BHIBA significantly induced the expression of brown adipocytes-related genes, including Ucp1, Cidea and Cpt1β, by activating the downstream mammalian target of rapamycin (mTOR) signal, thereby promoting the browning of white adipocytes. In addition, it has been pointed out that the effect of ketone bodies on WAT is realized by regulating the ratio of NADH/NAD^+^ [28]. BHIBA increases the ratio of NADH/NAD^+^, and up-regulation of NADH/NAD^+^ leads to redox pressure. In response to redox pressure, Ucp1 expression is increased, which can help deplete NADH and relieve redox pressure, a process that eventually mediates the browning of WAT. Moreover, it has been pointed out that BHIBA can be absorbed by WAT through MCT [25]. BHIBA levels are known to be elevated after prolonged exercise and during the post-exercise recovery period [29,30]. Therefore, we can hypothesize that exercise can lead to the decomposition of fatty acids to produce BHIBA and BHIBA promotes the expression of Ucp1 by activating the mTOR signal or by increasing the ratio of NADH/NAD^+^, thus promoting the browning of WAT.

#### 3.2.4. KYNA

In addition to the metabolites described above, exercise also promotes the release of KYNA from exercising skeletal muscles. One experiment [31], demonstrated that increased circulation of KYNA activates Gpr35 on adipocytes. Activation of Gpr35 stimulated the browning of WAT. In addition, Gpr35 can counteract inflammation induced by a high-fat diet. Deletion of Gpr35 inhibits exercise-mediated browning of WAT.

The above studies indicate that exercise can mediate the browning of WAT by regulating metabolites such as lactate, ketone bodies, succinate and KYNA. Therefore, metabolites play an important role in exercise-mediated browning of WAT.

### 3.3. Nervous System

**Figure 4:**
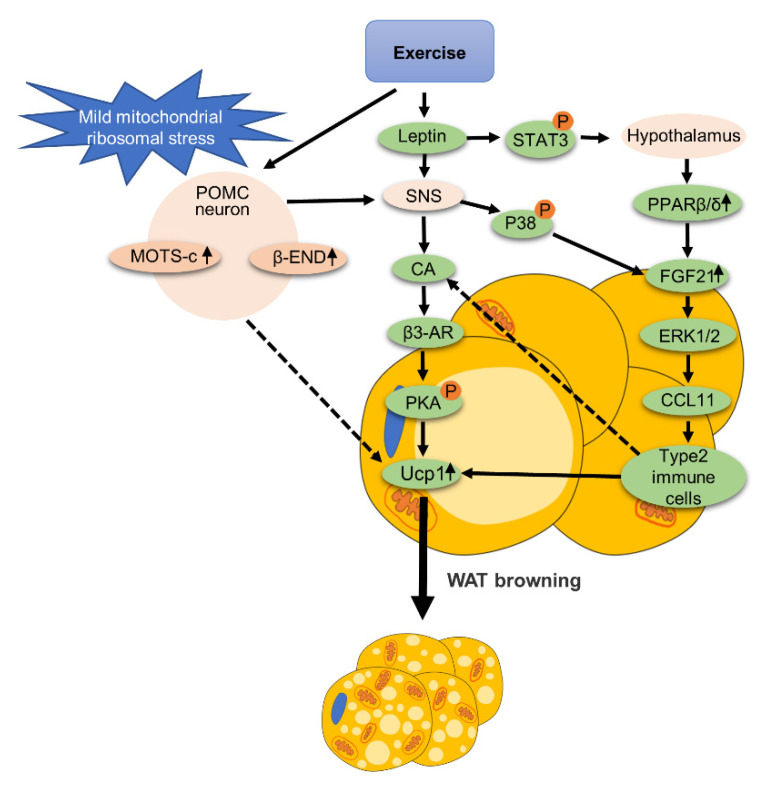
The role of the nervous system in exercise-mediated WAT browning. Exercise can increase the expression of β-endorphin (β-END) and the mitochondrial open reading frame of the 12S rRNA-c (MOTS-c) in hypothalamic proopiomelanocortin (POMC) neurons, and mediate WAT browning. POMC neurons can stimulate SNS to mediate the browning of WAT. Exercise enhances leptin signaling in the nervous system, which can also activate SNS. After SNS is activated, it stimulates the release of catecholamines (CA). After the release of CA, it acts on β3-adrenergic receptors (β3-AR) on adipocytes in WAT to stimulate PKA phosphorylation, thereby promoting the expression of Ucp1. In addition, leptin can stimulate phosphorylation of STAT3 in the hypothalamus to stimulate PPARβ/δ in eWAT, leading to the increased expression of FGF21. SNS activation also increases FGF21 expression by stimulating P38 signaling in WAT. The increased expression of FGF21 in adipocytes can activate ERK1/2 to promote the expression and secretion of the chemoattractant C-motif chemokine Ligand 11 (CCL11). CCL11 increases the level of type 2 immune cells in WAT. Type 2 immune cells are reported to stimulate CA release in WAT, which may be a possible mechanism for WAT browning.

The nervous system is involved in the exercise-mediated browning of WAT, as is summarized in Figure 4. An experiment [32], explains the relationship between exercise-mediated browning of WAT and proopiomelanocortin (POMC) neurons. It was shown that inducing mild mitochondrial ribosomal stress in POMC neurons, as indicated by the elevated expression of β-endorphin (β-END) and the mitochondrial open reading frame of the 12S rRNA-c (MOTS-c), would cause a series of beneficial metabolic effects. After POMC neurons were stressed, the mRNA expression levels of Cidea1 and Ucp1 in iWAT were significantly increased. The researchers demonstrated that exercise is a physiological condition that stimulates mild mitochondrial ribosomal stress in POMC neurons. In this experiment, mice were subjected to a running program for two weeks. The running speed was 13 m per minute for 50 min a day. The results showed that moderate-intensity exercise can increase mild mitochondrial ribosomal stress of POMC neurons in the hypothalamus, which is involved in promoting the browning of iWAT. In addition, the authors also mentioned that POMC neurons with mild mitochondrial ribosomal stress may promote innervation of the SNS to stimulate WAT heat production and browning.

Leptin is a hormone produced by adipose tissue that can act on the brain. Exercise can enhance leptin signaling [33]. After 12 weeks of VWR, the phosphorylation of signal transduction and the activator of transcription 3 (STAT3), which is a downstream effector of the leptin signaling pathway, was induced in the hypothalamus. In addition, VWR improved glucose and insulin sensitivity and reduced weight gain in mice fed with high fat diet (HFD). These results suggest that exercise can enhance the central leptin signal that could facilitate the improvement of the metabolic phenotype in HFD mice. The data above are consistent with the notion that exercise can improve leptin signaling in the brain [34]. In another study, exercise was shown to restore hypothalamic leptin signaling and insulin signaling and to reduce hypothalamic inflammation. Hypothalamic leptin and insulin signaling can cooperate with each other in POMC neurons to promote WAT browning [35]. Of note, one study [36] showed that leptin injection into the lateral ventricle regulates FGF21 expression in eWAT through PPARβ/δ, which mediates the browning of WAT. Leptin treatment resulted in the increased expression of Ucp1 protein in eWAT, as well as increased beige adipocyte markers such as Prdm16. In addition, leptin can also stimulate adipose decomposition through sympathetic neurons and mediate the browning of WAT. Experiments [37] confirmed that leptin can activate sympathetic neurons to promote fat decomposition. After SNS activation, WAT catecholamine release was stimulated. Then, catecholamines (CA) acted on β3-adrenalin receptors on adipocytes, stimulated PKA phosphorylation and promoted the expression of Ucp1. In addition, it was found that SNS activation increased the expression level of FGF21 in scWAT by stimulating P38 MAPK signaling. FGF21 binds receptor FGF receptor-1 (FGFR1) and B-Klotho (KLB) to form a complex on the surface of white adipocytes to promote the secretion of the chemoattractant C-motif chemokine Ligand 11 (CCL11) through activating ERK1/2 [38]. The secretion of CCL11 promotes the recruitment of type II immune cells in scWAT. It is reported that the type II immune response can stimulate the production and release of CA, thereby promoting heat production and the browning of WAT [39]. Therefore, exercise can enhance central leptin signaling which promotes the browning of WAT.

The above studies indicate that the central nervous system and peripheral sympathetic nervous system play important roles in the exercise-mediated browning of WAT. A previous review provides more detailed information about the role of the nervous system in the browning of WAT induced by exercise [34].

### 3.4. Exerkines

**Figure 5:**
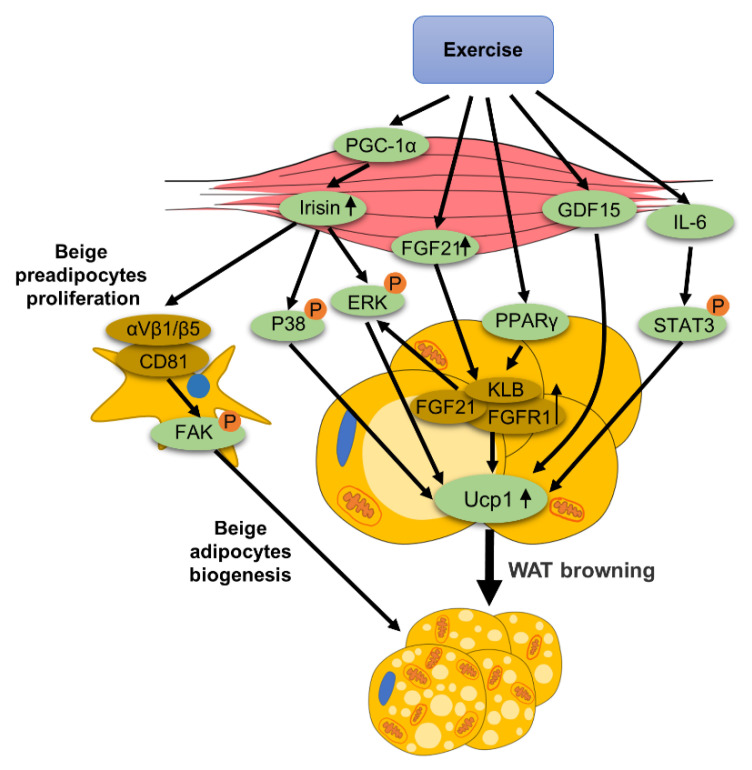
The role of exerkines in exercise-mediated WAT browning: exercise increases irisin expression through PGC-1α. CD81 integrates with αV-β1 and αV- /β5 to form a complex in beige preadipocytes. Irisin binds to this receptor to promote the focal adhesion kinase (FAK) phosphorylation, thereby facilitating the proliferation of beige preadipocytes. In addition, irisin mediates the browning of WAT through P38 and ERK MAP kinase signals in adipocytes. Exercise enhances the expression of FGF21, and the binding of FGF21 to its receptor FGF receptor-1 (FGFR1) and B-Klotho (KLB) promotes the expression of Ucp1. In addition, peroxisome proliferator-activated receptor γ (PPARγ) in eWAT can induce WAT to express KLB. Exercise induces an increase in serum IL-6 levels, thereby increasing adipose tissue IL-6. IL-6 enhances Ucp1 expression by stimulating the phosphorylation of signal transduction and activator of transcription 3 (STAT3). Exercise promotes the expression and secretion of growth differentiation factor 15 (GDF15), which facilitates WAT browning.

Exercise can mediate the production and release of many exerkines, such as FGF21, IL-6, irisin, meteorin-like (METRNL), myostatin (MSTN), follistatin and growth differentiation factor 15(GDF15). The roles of some exerkines in WAT browning is summarized in Figure 5.

#### 3.4.1. FGF21

Both cold exposure and β3-adrenergic stimulation strongly induce FGF21 expression in scWAT through cyclic adenylate mediated activation of protein kinase A and P38 MAPK, suggesting that adipose FGF21 is a downstream effector of sympathetic activation. After specific overexpression of FGF21 in adipocytes (AD-F21 Tg), the expression of Dio2, Dusp4 and Ucp1 tended to be up-regulated, which are marker genes of WAT browning [40]. Mice with adipose tissue-specific knockout of FGF21 (A-FGF21KO) were subjected to 6-day cold exposure [38]. The results showed that the temperature in the groin area of A-FGF21KO mice decreased more significantly after cold exposure compared with the control group, indicating that the cold-induced thermogenesis of scWAT of A-FGF21KO mice was impaired. Notably, after chronic cold exposure, mRNA levels of key genes such as Ucp1, Cidea, Elovl3 and Cpt1β in scWAT of A-FGF21KO mice were significantly lower than control mice, as was the protein expression of Ucp1. These results indicate that adipose tissue FGF21 is necessary for the browning of WAT and thermogenesis. As mentioned above, FGF21 binds FGFR1 and KLB to form a complex. ERK1/2 promotes the secretion of CCL11 and mediates WAT browning. Mohammad Abu-Odeh et al. [40] studied the role of KLB by using adipose-specific KLB KO (ABKO) mice. WT and ABKO mice were treated with CL-316,243 daily injections for one week. Ucp1 protein expression was significantly up-regulated after treatment with CL-316,243 in WT mice, and this up-regulation was abolished in ABKO mice. These results indicate that KLB is required for FGF21 to mediate the browning of adipocytes.

It is worth noting that exercise can increase FGF21 secretion and elevate plasma FGF21 levels [41]. Meanwhile, exercise can induce the expression of FGFR1 and KLB in WAT through transcriptional activation, mediated by peroxisome proliferator-activated receptor PPARγ [42]. Treadmill exercise for 4 weeks promoted the activity of PPARγ in eWAT of mice. After treatment with the a PPARγ inhibitor the up-regulation of FGFR1 and KLB in adipose tissue, induced by exercise, was significantly inhibited. In conclusion, FGF21 signaling is an important pathway in exercise-mediated browning of WAT.

#### 3.4.2. IL-6

A recent paper [43], has demonstrated that peripheral IL-6 promotes the browning of WAT. In this study, IL-6 promoted the browning of WAT in an autocrine/paracrine manner. It was further confirmed that peripheral IL-6 mediated the browning of WAT by stimulating STAT3 Tyr705 phosphorylation to enhance the transcription of PPARγ and Ucp1. In addition, they produced IL-6-KO mice and found that IL-6-KO mice reduced the browning of WAT by inhibiting STAT3 phosphorylation, accompanied by disturbed glucose homeostasis and accelerated hepatic steatosis. This indicates that IL-6 is very important for the browning of WAT. Another paper [11], also demonstrated that the secretion of IL-6 from adipose tissue can activate STAT3 and inhibit glucose production in the liver. In addition, Tong Liu et al. [44] extracted fat from scWAT in the thighs or abdomen of five patients and transplanted it into the dorsal side of 6- to 8-week-old female nude mice. They found that the browning of WAT induced by fat transfer was parallel to the level of IL-6 and that elevated IL-6 promoted the browning of WAT.

Exercise also has a significant effect on the elevation of serum IL-6. Ali Gorzi et al. [45] tested the difference in serum IL-6 in Wistar rats after different cycles of training including high-intensity interval training (HIIT) training compared with a control group of non-training rats. Two cycles of HIIT were used in this experiment, namely 4-day HIIT and 7-day HIIT. The scheme for 4-day HIIT was as follows: 3 days of exercise and 1 day of rest and 7 cycles of training. The scheme for 7-Day HIIT was as follows: 6 days of exercise and 1 day of rest and 4 cycles of training. The results showed that serum IL-6 levels were higher after both 4 and 7-day HIIT training than in the control group and that serum IL-6 levels were higher after 7-day HIIT than after 4-day HIIT. In addition, Phureephat Larsuphrom et al. [46] reviewed 10 years of studies and concluded that serum IL-6 increased significantly after resistance exercise and aerobic endurance exercise. In summary, HIIT, resistance exercise and endurance exercise all increased serum IL-6 levels. Therefore, exercise induces the up-regulation of IL-6 to mediate the browning of WAT.

#### 3.4.3. Fndc5/Irisin

As previously described, exercise induces cleavage of the Fndc5-containing domain in skeletal muscle. Fndc5 cleavage produces irisin, which is secreted into circulation. Irisin mediates the browning of WAT through the P38 and ERK MAP kinase signal. In addition, irisin activation of the focal adhesion kinase (FAK) signaling pathway can promote the biogenesis of beige adipocytes [47]. It was shown that irisin promotes the proliferation of beige adipocyte progenitor cells (APCs) through the CD81/integrin receptor-mediated activation of FAK pathways, leading to the formation of beige adipocytes. This beige APCs population is proliferative and is marked by cell-surface proteins, including CD81. CD81^+^APCs proliferate highly in iWAT, and CD81 integrates with αV-β1 and αV-β5 to form a complex. Irisin binds to this complex to activate the FAK signal, mediating the formation of beige fat cells. Importantly, CD81 loss can lead to diet-related obesity, insulin resistance and adipose tissue inflammation. Therefore, exercise up-regulates irisin to induce the browning of WAT, and whether irisin-mediated CD81-positive APCs proliferation and beige adipogenesis is involved in the above process needs further investigation.

Leptin, as mentioned above, can also mediate the browning of WAT through regulating the nervous system. In addition, exerkines such as METRNL, MSTN, follistatin, and GDF15 have also been shown to mediate the browning of WAT and have been described in detail in other review articles [48,49]. In conclusion, exerkines are important effectors in exercise-mediated browning of WAT.

### 3.5. Lipolysis

**Figure 6:**
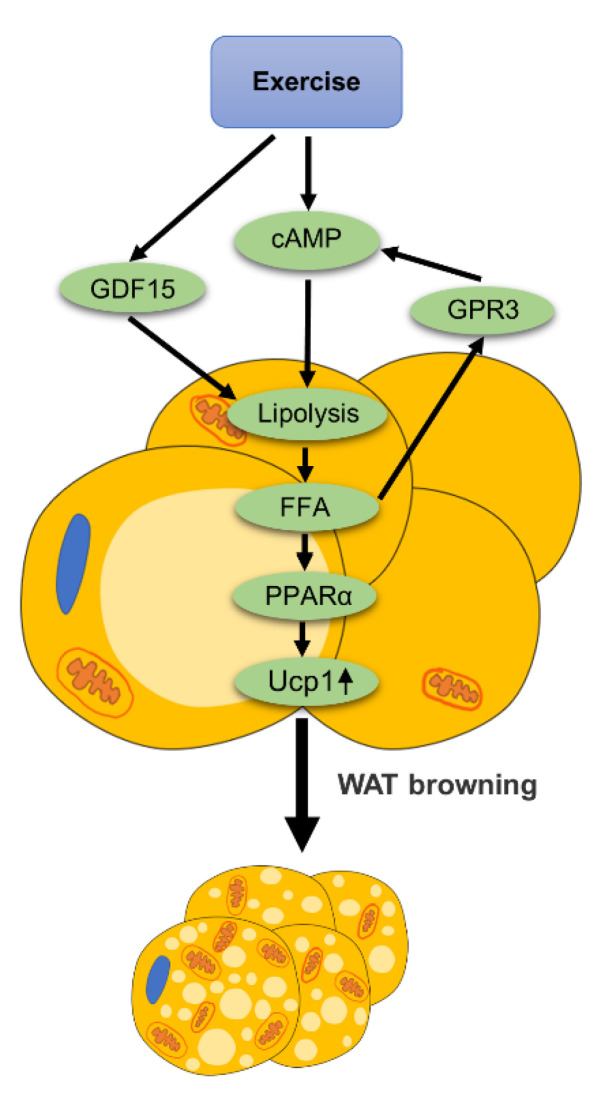
The role of lipolysis in exercise-mediated WAT browning: exercise increases the level of cAMP in adipocytes to promote lipolysis and produce free fatty acids (FFA). Exercise promotes lipolysis of adipose tissue by up-regulating the expression of GDF15. The lipolysis signal directly activates GPR3, thereby promoting the browning of WAT via the cAMP signal. In addition, FFA produced by lipolysis can act as ligands and activates PPARα. Activation of PPARα promotes the expression of thermogenic genes such as Ucp1 and mediates the browning of WAT.

Exercise can promote lipolysis. It is worth noting that lipolysis signals can mediate the browning of WAT, as summarized in Figure 6.

As early as 2011, [50] found that adipose tissue-specific knockout of desnutrin resulted in decreased expression of fatty acid oxidation (FAO) genes and thermogenic genes. It was also found that FAO was blunted in WAT. Desnutrin is a key hydrolytic enzyme for the hydrolysis of triacylglycerol (TAG) to diacylglycerol (DAG). Adipose-specific desnutrin knockout mice (desnutrin-ASKO mice) were studied and it was found that both Ucp1 mRNA and protein expression were reduced in these mice. In addition, the BAT of desnutrin-ASKO mice showed larger lipid droplets, reduced mitochondria number, and most of the mitochondria were composed of randomly directed cristae, which are all characteristics of WAT. This suggests that the BAT of these mice is transformed to a WAT-like pheotype, which is due to inhibition of lipolysis.

Attention has been aroused by a recent experiment [51]. The experiment proposed that the relationship between lipolysis and WAT browning may be attributed to the existence of G protein-coupled receptor 3 (GPR3). GPR3 does not require ligand activation, and when GPR3 integrates into the cell membrane, it can automatically activate downstream signals. The experiment pointed out that the high expression of GPR3 in WAT will promote the browning of WAT. Interestingly, the lipolysis signal directly promotes the expression of GPR3, thereby promoting thermogenesis. Most importantly, this has also been confirmed in human experiments. Increasing the level of GPR3 in human subcutaneous white adipocytes induced the expression of thermogenesis gene Ucp1, mitochondrial respiration and fatty acid uptake. Therefore, the lipolysis signal directly promotes the expression of GPR3, thereby promoting the expression of Ucp1 genes and proteins and facilitating WAT browning. In addition, free fatty acids (FFAs) produced by lipolysis can act as ligands of PPARα. Once activated by FFAs, PPARα promotes the expression of thermogenic genes such as Ucp1. As a result, a conjecture is proposed: exercise promotes lipolysis, which triggers a GPR3 signal or produces FFAs to activate PPARα, thereby promoting the browning of WAT. This may be a possible mechanism underlying exercise-induced WAT browning and merits further investigation.

Claire Laurens et al. [52] conducted conditional medium (CM) experiments on adipocytes and found that CM from skeletal muscle cells of acute high-intensity and chronic moderate-intensity exercise increased the basic lipolysis of human adipocytes. In this study, they established an acute high-intensity exercise model and chronic moderate-intensity exercise training model using electrical impulse stimulation of human muscle ducts to induce forced contraction. The results showed that exercise-mediated skeletal muscle contraction produced exerkines that activated adipocyte lipolysis in vitro. They then performed proteomic screening of skeletal muscle cells and determined that GDF15 was significantly up-regulated in CM in both models. In addition, they found that the GDF15 homologous receptor GFRAL was highly expressed in the whole adipose tissue and in isolated adipocytes. Importantly, this study indicated that GDF15 significantly increased lipolysis. Therefore, exercise can promote lipolysis of adipose tissue by up-regulating GDF15 expression. Another experiment [53] showed that the browning of WAT was inhibited upon GDF15 knockout. In this study, GDF15 knockout was performed in mice, and Ucp1mRNA was significantly reduced in scWAT, and the protein expression of Ucp1 could not be detected. Therefore, we hypothesized that exercise promotes the lipolysis of adipose tissue partially by increasing the expression of GDF15, resulting in the browning of WAT. In conclusion, the studies above demonstrate the functional role of lipolysis in exercise-mediated browning of WAT.

## 4. Effects of Different Exercise Programs on Promoting the Browning of WAT

**Figure 7:**
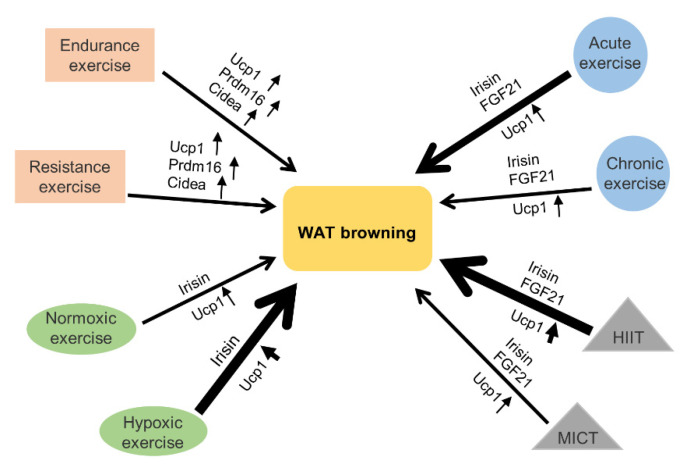
Effects of different exercise programs on the browning of WAT: various types of exercise, including high-intensity interval training (HIIT), moderate intensity continuous training (MICT), acute exercise, chronic exercise, normoxic exercise, hypoxic exercise, aerobic exercise and resistance exercise can mediate the browning of WAT. Among them, HIIT can promote WAT browning more effectively than MICT. Acute exercise can promote WAT browning more effectively than chronic exercise. Hypoxic exercise can promote WAT browning more effectively than normoxic exercise. The expression levels of browning-related genes, such as Ucp1, Prdm16 and Cidea are up-regulated in WAT after many different types of exercise. Irisin and FGF21 are involved in promoting WAT browning and are mediated by many different types of exercise. The thickness of the arrow indicates the strength of the effect exerted by exercise. The thicker the arrow is, the stronger the effect is.

Different types of exercise had different effects on the browning of WAT. We summarize various types of exercise to reveal that effectiveness of exercise-mediated browning of WAT is related to exercise styles, intensity, duration and availability of oxygen during exercise (Figure 7). Based on different classification standards, exercise can be categorized into different types, HIIT and moderate intensity continuous training (MICT), acute exercise and chronic exercise, hypoxic exercise and normoxic exercise, endurance exercise and resistance exercise (Figure 7).

### 4.1. HIIT and MICT

HIIT needs to be carried out for short periods, above the lactate threshold and close to Vo_2max_ and punctuated with light workouts or rest to achieve the best results. HIIT is the best way for people to achieve the best results in this fast-paced environment because most people do not have enough time for exercise. MICT lasts longer and has a moderate intensity of training.

It was found in [54] that HIIT can effectively reduce body weight and increase energy expenditure. HIIT also enhanced mitochondrial biogenesis and β-oxidation in scWAT, playing an important role in improving metabolism. Importantly, HIIT increased the expression of Fncd5 and Ucp1, mediating the browning of scWAT. Mousa Khalafi et al. [55] compared the effects of HIIT and MICT on the browning of scWAT and found that HIIT had a greater effect on the browning of scWAT than MICT. The experimental HIIT regimen consisted of running at 85 to 90 % of maximum speed for four minutes, followed by two minutes at 50 % of maximum speed. In this way, the running speed of male adult Wistar rats increased from 17 m/min in the first week to 26 m/min in the 10th week. For the next 2 weeks, the running speed was kept at 26 m/min. The MICT exercise regimen is to keep the running speed at 65% to 70% of the maximum speed. The running speed of the rats increased from 12 m/min in the first week to 16m/min in the 10th week and was maintained at 16 m/min for the next two weeks. The results showed that both exercise trainings led to an increase in irisin and FGF21. As mentioned above, both irisin and FGF21 can induce the browning of WAT. Subsequently, the researchers detected that both types of exercise increased Ucp1 protein expression. Compared with MICT, HIIT has a greater impact on the above changes. In addition, both HIIT and MICT reduced the abundance of C/EBP-α and C/EBP-β in subcutaneous adipose tissue, which are biomarkers of adipogenesis. In conclusion, the effect of exercise-mediated browning of WAT is related to exercise intensity, and HIIT is better than MICT in inducing the browning of WAT.

### 4.2. Acute Exercise and Chronic Exercise

Acute exercise refers to one-time exercise, while chronic exercise refers to repeated long-term exercise. Both of them can mediate the browning of WAT. Some experiments proved that acute exercise had a significant effect on the browning of WAT. Shen Y et al. [56] demonstrated that acute exercise inhibited adipogenic gene expression and may modulate thermogenesis through activation of PGC-1α and Ucp1 in WAT. In this study, the expression of PGC-1α was significantly increased in eWAT and iWAT after a 120-min round of swimming in mice, and Ucp1 was significantly up-regulated in eWAT. Therefore, acute exercise may regulate the expression of PGC-1α and Ucp1 to achieve browning of WAT. Eunhee C et al. [57] showed that 90 min of acute swimming exercise could lead to increased expression of the Fndc5 gene and protein in soleus muscle and increased Fndc5 protein in gastrocnemius muscle. Consistently, the protein expression of Ucp1 was increased in scWAT. Studying the half-life of exercise-inducing factors will help us better understand the mechanism of exercise-mediated WAT browning.

In addition, acute exercise-induced browning of WAT was also associated with FGF21. Yuko Tanimura [58] and others pointed out that acute exercise can increase the expression of FGF21. In this experiment, mice were subjected to a 30m/min treadmill exercise for 60 min. It was found that after exercise training, FGF21 in the serum and skeletal muscle of mice increased significantly. In mice, the role of FGF21 in inducing browning of WAT has also been confirmed. In short, this suggests that the induction of FGF21 may be involved in acute exercise-induced WAT browning.

Some studies showed that chronic exercise could also promote the browning of human scWAT. As mentioned earlier, 4 weeks of running may increase the activity of PPARγ in eWAT, which can up-regulate FGFR1 and KLB in adipose tissue and mediate the browning of eWAT [42]. This suggests that FGF21 signaling is involved in promoting chronic exercise-mediated browning of WAT. In addition, chronic exercise can also mediate the browning of WAT by elevating the level of irisin in the blood. After 3 weeks of free-wheeling exercise on mice and 10 weeks of endurance exercise on healthy people, the levels of irisin in the blood of mice and humans increased [7]. Experimenters [59] trained sedentary people with different BMIs (all non-diabetic) on bicycles for 12 weeks. The exercise intensity was 70% to 80% of the maximum heart rate, three times a week. It was found that the mRNA levels of Ucp1 and Cpt1β in scWAT were increased in all participants after exercise. However, the gene and protein expressions of Ucp1 were statistically different only in obese women. The beige adipose tissue marker TBX1 also increased significantly after the exercise.

Another experiment [60] proved that the Ucp1 gene in the iWAT of obese animals under thermoneutral conditions was missing and was not induced by exercise training. Mice at 12-weeks-old that were fed a high-fat diet in a thermoneutral environment were subjected to swimming training for 4 weeks (hour a day, 5 days a week). After training, although PGC-1α, Dio2 and PPARα were up-regulated, the Ucp1 gene was not detected in mouse iWAT, and exercise training did not induce Ucp1 gene expression. This may be due to the suppression of sympathetic nerve excitation under thermal neutral conditions. Activation of the sympathetic nerve plays an essential role in promoting WAT browning and cold environment is important for the activation of sympathetic nerve. When it is thermally neutral, sympathetic nerve excitation is blunted. As a result, exercise-induced WAT browning is impaired under thermoneutral conditions. Thus, appropriate temperature could be important for the WAT browning mediated by chronic exercise.

### 4.3. Normoxic Exercise and Hypoxic Exercise

A large number of experiments have proved that hypoxic exercise is better than normoxic exercise in reducing obesity. However, there are relatively few studies on the browning of WAT by exercise under hypoxic conditions. One study [61] examined the adipose tissue changes in patients with type 1 diabetes after moderate intensity (50% lactate threshold, 40 min) exercise under normoxia or hypoxia. It was found that the serum irisin level was higher after exercise under hypoxia than under normal oxygen. The increase of irisin is closely related to the browning of WAT. In addition, according to Yingli Liu et al. [62], the up-regulation of leptin in vWAT induced by exercise in a hypoxic environment was greater than that in a normoxic environment. Leptin can enhance browning of WAT via regulating the nervous system (Figure 4). Thus, based on the above result, it is hypothesized that hypoxic exercise may promote WAT browning more effectively than normoxic exercise. In addition, we all know that hypoxic exercise is better than normoxic exercise for weight loss, is this because of the enhanced browning of WAT? In this regard, we propose a conjecture that hypoxia exercise has a better effect on promoting browning of WAT than normoxia exercise, which may explain why it is more effective in reducing obesity and ameliorating metabolic disorders. Further experiments are needed to verify the above hypothesis.

### 4.4. Endurance Exercise and Resistance Exercise

It is reported that endurance exercise and resistance exercise similarly induced browning of iWAT and retroperitoneal WAT (rpWAT). Caroline de Carvalho Picoli et al. [63] compared the effect of endurance exercise (running) and resistance exercise (ladder climbing) on WAT browning. The running plan was as follows: the exercise intensity was 70% of the maximum speed, and the slope was 30–40 min in the first week, 35–50 min in the second week, 50–60 min in the third week, and 60 min in week 4 until week 8. This training pattern was for endurance exercise. The ladder climbing scheme was as follows: loads were 50%, 75%, 90% and 100% of body weight, respectively, five times a week, lasting for eight weeks. This training pattern was for resistance exercise. The results showed that the protein expression of Ucp1 in iWAT and rpWAT in mice after the two exercise regimens was higher than that in sedentary mice. In addition, both kinds of training increased mRNA expression of Ucp1, PGC-1α, Prdm16 and Cidea in iWAT and rpWAT. In conclusion, both endurance training and resistance training can induce browning of WAT in mice. In human studies, however, the case was different. Resistance exercise could not change the level of irisin in human serum, suggesting that resistance exercise may not cause the browning of WAT in humans [64]. In addition, for obese people, the combination of endurance and resistance training had no effect on the browning of WAT. R. Stinkens et al. [65] conducted a 12-week progressive, combinatory exercise training program for men with good metabolism or obesity. The subcutaneous fat tissue was taken 72 h after the last round of exercise. After an overnight fast, a biopsy of abdominal subcutaneous fat tissue (about 1 g) was collected from 6–8 cm outside the umbilicus. However, the ambient temperature for the experiment was not mentioned in the paper. The results showed that there were no significant changes in gene expression of browning markers Cidea, Prdm16 and PGC-1α in scWAT after exercise training, and no Ucp1 mRNA was detected. Therefore, the results above suggest that a combination of endurance exercise and resistance exercise do not mediate the browning of WAT in humans.

Regarding the above negative results in humans, we propose two conjectures: the first is the choice of ambient temperature. Cold exposure alone can activate SNS to induce the browning of WAT. Exercise, combined with cold stimulation, may increase sympathetic nerve excitement better, thereby inducing WAT browning more efficiently. Therefore, in the selection of exercise programs, temperature factors could be taken into consideration. For example, choosing a swimming program, or exercising in a cooler environment may be more efficient to induce the browning of WAT in human. The second point is human testing methods for the browning of WAT. In previous experiments, the invasive approach was often chosen, which is a stimulus that can have other unforeseen effects on the body. Besides this, experiments on animals suggest that the browning of WAT in different parts is different (see the discussion in the next section). These suggest that detection methods and sampling parts of the adipose tissue may influence the accuracy of the results. Thus, human experiments for the study of exercise-mediated WAT browning need to be improved accordingly.

As described above, the effectiveness of exercise-mediated WAT browning depends on the type of exercise. It should be noted that for some special individuals, the choice of exercise styles may need to be carefully considered. For example, cachexia is a disease characterized by energy waste and muscle loss. Appropriate exercise helps to improve cachexia and muscle loss. However, cachexia is usually associated with WAT browning, which could aggravate cachexia and muscle loss through increasing energy expenditure and promoting lipolysis [66]. Therefore, for the treatment of cachexia, it is suggested to choose a mild exercise style that may not effectively induce WAT browning, so as to avoid the adverse effects of WAT browning on cachexia.

## 5. Reponses to Exercise-Mediated Browning Vary in Different Anatomical Parts of Adipose Tissue

**Figure 8:**
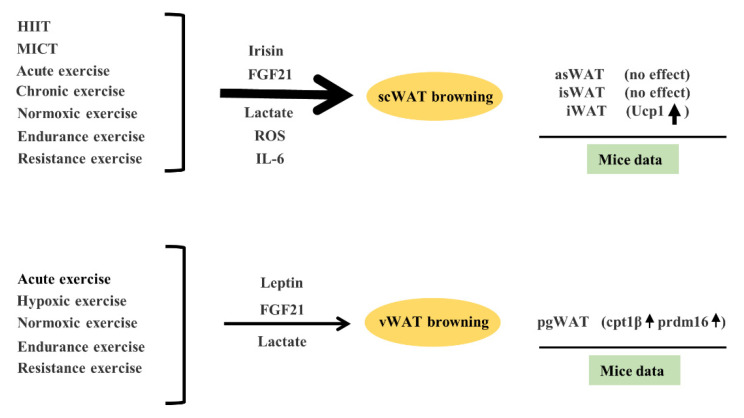
Exercise-mediated browning effectiveness varies in different anatomical parts of adipose tissue. Exercise, including HIIT, MICT, acute exercise, chronic exercise, hypoxic exercise, normoxic exercise, endurance exercise and resistance exercise, can promote the browning of subcutaneous white adipose tissue (scWAT), which is mainly mediated by exercise-induced up-regulation of irisin, ROS, FGF21, IL-6 and lactate. As suggested in the mice experiment, exercise had no effect on the browning of anterior subcutaneous WAT (asWAT) and interscapular WAT (isWAT) but can increase the expression of Ucp1 in iWAT. Exercise, including acute exercise, hypoxic exercise, normoxic exercise, endurance exercise and resistance exercise can promote the browning of visceral white adipose tissue (vWAT), which is mainly mediated by exercise-induced up-regulation of leptin signal, FGF21 and lactate level. As suggested in the mice experiment, exercise increased cpt1β and prdm16 expression in perigonadal WAT (pgWAT). The thicker the arrow is, the stronger the effect is.

Studies have shown that adipose tissue in different parts of the body could have different browning effectiveness in response to exercise (Figure 8). As mentioned above, both HIIT and MICT can induce the browning of scWAT. Acute exercise mediated the browning of scWAT and vWAT. In contrast, chronic exercise mediated browning in scWAT rather than vWAT. Hypoxic exercise may mediate browning in vWAT, which is more difficult to brown than scWAT and normoxic exercise can mediate scWAT and vWAT browning. In addition, both endurance exercise and resistance exercise can mediate scWAT and vWAT browning. Adam C et al. [67] studied the responses of different adipose tissue depots to exercise in mice. In the scWAT of mice, exercise training did not affect the expression of the beige gene in anterior subcutaneous WAT (asWAT) and interscapular WAT (isWAT), but it increased the expression of the Ucp1 gene and protein in iWAT. In the visceral adipose tissue of mice, exercise training increased Cpt1β and Prdm16 gene expression in perigonadal WAT (pgWAT) but did not affect Ucp1 or Cidea.

The potential mechanism for the different responsiveness of different anatomical parts of adipose tissue to exercise-mediated WAT browning is still unclear. The various responses of different anatomical adipose depots to exercise-mediated changes in ROS, metabolites, nervous system, exerkines and lipolysis could be the underlying mechanism, which needs further investigation. Furthermore, different parts of WAT may respond to exercise-mediated browning of WAT via different pathways. During exercise, such as HIIT and MICT, irisin and FGF21 were up-regulated, which could promote the expression of Ucp1 and facilitates scWAT browning [55]. Exercise-mediated scWAT browning could be attributable to irisin-triggered-activation of P38 and ERK signaling [9]. In addition, browning of scWAT may also be related to other substances such as ROS [15], IL-6 [44], and so on. Leptin may be involved in the exercise-mediated browning of eWAT. Leptin-mediated up-regulation of PPARβ/δ expression induced increased expression of FGF21 in eWAT, resulting in browning of eWAT [36]. In addition, lactate produced by exercise could mediate both scWAT and vWAT browning [23,24].

The ideas above have important implications for effective and site-specific fat loss demanded by different individuals. For example, excessive accumulation of vWAT can lead to organ disease, inflammation and other adverse health conditions. Reducing the accumulation of vWAT may be critical for people with health problems caused by excessive accumulation of vWAT or those with beer bellies. The browning of vWAT can reduce the accumulation of vWAT. Therefore, it is of great significance to explore a better exercise program to facilitate vWAT browning for this group of people. The scWAT has beneficial effects on the body such as keeping warm and cushioning. However, for those looking for a lower body fat percentage and a better silhouette, reducing scWAT could be desirable. Therefore, according to the different browning effectiveness of WAT in different anatomical parts in response to exercise, the exercise mode could be developed and selected to achieve the browning of WAT in different parts.

## 6. Conclusions

Growing evidence shows that exercise can induce the browning of WAT. Multiple factors, including ROS, metabolites, nervous system, exerkines and lipolysis can facilitate exercise-mediated browning of WAT. In-depth understanding of the mechanisms underlying exercise-mediated browning of WAT would provide new insight into the beneficial effects of exercise on metabolic health. Based on our review, it is suggested that HIIT and hypoxic exercise are the most recommended for the treatment of obesity, through the browning of the WAT. However, in the selection of exercise types, intensity and duration, other factors, such as the individual’s physiological and pathophysiological conditions, should be considered so as to yield a safe and effective outcome of exercise. It should be noted that the browning effectiveness of WAT varies depending on the styles of exercise and the response of anatomically different fat depots to exercise. Therefore, the development of effective exercise strategies for WAT browning, according to different groups of people or different needs for fat loss, is worth further exploration. In conclusion, the study and application of exercise-mediated browning of WAT is of great help and promising for the improvement of metabolism and the fight against obesity.

## Data Availability

Not applicable.
